# Synthesis and Antitumor Activity of 3-Methyl-4-oxo-3,4-dihydroimidazo [5,1-*d*][1,2,3,5]tetrazine-8-carboxylates and -carboxamides

**DOI:** 10.3390/molecules15129427

**Published:** 2010-12-20

**Authors:** Dan Liu, Jian-Guo Yang, Jie Cheng, Lin-Xiang Zhao

**Affiliations:** 1 Key Laboratory of Structure-Based Drugs Design & Discovery of Ministry of Education, Shenyang Pharmaceutical University, Shenyang 110016, China; E-Mails: sammyld@163.com (D.L.); spujack2008@126.com (J.C.); 2 Zhejiang Jinhua CONBA Bio-pharm Co. Ltd., Jinhua 321016, China; E-Mail: yangjg999@163.com (J.-G.Y.)

**Keywords:** antitumour activity, 3-methyl-4-oxo-3,4-dihydroimidazo[5,1-*d*][1,2,3,5]-tetrazine-8-carboxylates, 3-methyl-4-oxo-3,4-dihydroimidazo[5,1-*d*][1,2,3,5]tetrazine-8-carboxamides, synthesis

## Abstract

Seventeen novel 3-methyl-4-oxo-3,4-dihydroimidazo[5,1-*d*][1,2,3,5]tetrazine-8-carboxylate and -carboxamide derivatives were synthesized and evaluated for their growth inhibition in seven human solid tumor and a human leukemia HL-60 cell lines. Compound **IVa** showed more activity than the other compounds and the positive control temozolomide. In the presence of 40 μg/mL of **IVa,** the survival rate of all tested tumor cells was less than 10%. Esters displayed more potent antitumour activity than amides and temozolomide against HL-60 cells. These compounds also exhibited considerably enhanced water-solubility.

## 1. Introduction

**Scheme 1:**
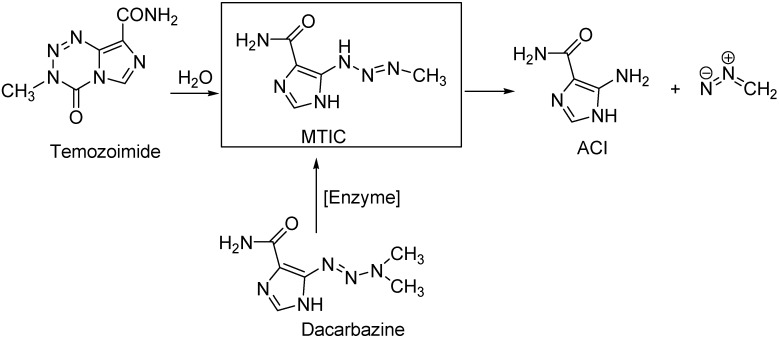
Metabolic pathways of temozoloimide and dacarbazine.

Temozolomide (Temodar, 3-methyl-4-oxo-3,4-dihydroimidazo[5,1-*d*][1,2,3,5]tetrazine-8-carbox-amide), was approved bythe U.S. FDA to treat the patients suffering from glioblastoma and anaplastic astrocytoma in 1999. *In vivo,* temozolomide is converted into 5-(3-methyl-1-triazeno)imidazole-4-carboxamide (MTIC) through chemical degradation without enzymatic catalysis, whereas dacarbazine requires metabolic activation to generate the active form [1]. MTIC decomposes spontaneously to form 5-aminoimidazole-4-carboxamide (ACI) and a methyldiazonium ion [2], and the latter attacks the guanine segment of a sequence of three or more guanines on DNA leading to DNA methylation of (Scheme 1). This DNA damage can be repaired by *O*-6-methylguanine-DNA methyltransferase (MGMT) expressed in some tumor cells, which is the primary mechanism of tumor resistance to alkylating agents, including temozolomide [3]. Currently, combination therapies of temozolomide with established anticancer drugs, such as cisplatin and irinotecan are being investigated [4,5].

We were interested in the imidazotetrazinone skeleton due to its particular metabolism and low toxicity. In order to improve the antitumor activity and water-solubility, some new temozolomide hydrochlorides were designed and synthesized and their antitumor activity was assayed. There is a general agreement that antitumor imidazotetrazinones display powerful activity when the N3 position is methyl-substituted [6,7], so two series of compounds **IIIa - IIIh** and **IVa - IVi** with a methyl group at the N3 position and esters or amides group at the 8 position were designed and synthesized. The basic substitutents at the position 8 formed hydrochlorides to improve the stability and solubility in water. The target compounds could be hydrolyzed *in vivo* to form 3-methyl-4-oxo-3,4-dihydro-imidazo[5,1-*d*][1,2,3,5]tetrazine-8-carboxylic acid, which is the active metabolite of temozolomide and shows moderate activity against TLX lymphoma *in vitro*. In this paper, the antitumor activity of the 17 new compounds against seven human solid tumor cell lines (PC-3, HCT-15, T47D, MDA-MB-231, DU145, HT29, and LNCaP) and a leukemia cell line (HL-60) was tested *in vitro*. Further antileukemia studies are also planned.

## 2. Results and Discussion

### 2.1. Chemistry

**Scheme 2:**
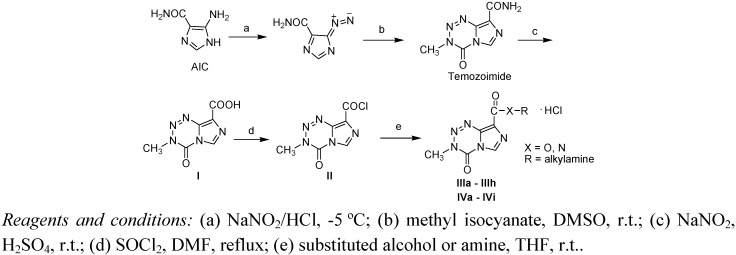
Synthetic route to the target compounds.

The synthetic route to the 3-methyl-4-oxo-3,4-dihydroimidazo[5,1-*d*][1,2,3,5]tetrazine-8-carbox-ylates **IIIa - IIIh** and 3-methyl-4-oxo-3,4-dihydroimidazo[5,1-*d*][1,2,3,5]tetrazine-8-carboxamides **IVa - IVi** is presented in Scheme 2. The substituents of compounds **IIIa - IIIh** and **IVa - IVi** are listed in Table 1. Commercially available 5-aminoimidazole-4-carboxamide (AIC) was first treated with sodium nitrite in dilute hydrochloric acid to give the corresponding diazo compound, to which methyl isocyanate was added dropwise to afford temozoimide [8]. After hydrolysis with sodium nitrite and sulfuric acid, carboxylic acid **I** was produced. Acyl chloride **II** provided by reaction of compound **I** and thionyl chloride was reacted with alcohols and amines to yield the target compounds **IIIa - IIIh** and **IVa – Ivi**, respectively.

### 2.2. Antiproliferative activities

In the present study, the antiproliferative activity of the synthesized compounds was tested *in vitro* on seven human tumor cell lines, including prostate cancer (PC-3, LNCaP and DU-145), breast cancer (T47D, MDA-MB-231) and colon cancer (HT-29, HCT-15) by the MTT assay. The potential cytotoxicities of all compounds were determined by measuring the percentage of cell survival, as summarized in Table 1. The data suggested that at the concentration of 40 μg/mL, the amide **IVa** displayed powerful inhibition against all seven tested tumor cell lines, whose viabilities were all below 10%. The other compounds and temozolomide showed moderate inhibition against all cell lines. Among the other compounds derivative **IVh** showed better inhibition against T47D breast cancer cells and DU145 prostate cancer cells (cell viability was 32.65% and 32.77% respectively), while the corresponding results for temozolomide were 62.35% and 70.67%. Compared with the control drug, compound **IVi** possessed more potent inhibitory activity against breast cancer cell T47D，the survival percentage was 34.97% *vs*. 62.35% of temozolomide. On the whole, the temozolomide analogues showed higher cytotoxicity against seven tested cancer cell lines when the position 8 of temozolomide was substituted by a acylamide.

**Table 1:** The substituents and cytotoxicity of the title compounds **IIIa** - **IVi**.

Compd.	X	R	Survival percent / %^a)^						
			PC-3	LNCaP	T47D	MDA-MB-231	DU145	HT29	HCT-15
**IIIa**	O	-CH_2_CH_2_N(CH_3_)_2_^.^ HCl	87.98±0.40	89.37±1.75	66.40±1.07	74.61±1.61	92.60±1.37	93.89±1.28	92.30±0.69
**III** **b**	O	-CH_2_CH_2_N(C_2_H_5_)_2_^.^ HCl	92.10±1.42	81.89±1.45	63.65±1.41	70.36±1.50	92.70±2.05	86.22±1.66	82.00±1.93
**III** **c**	O	-(CH_2_)_3_N(CH_3_)_2_^.^ HCl	114.86±0.52	91.33±0.94	81.13±0.35	76.51±1.03	90.15±1.70	79.92±1.59	95.59±0.49
**III** **d**	O	-(CH_2_)_3_N(C_2_H_5_)_2_^ .^ HCl	88.46±0.96	75.95±0.41	62.93±1.40	65.70±0.59	74.97±1.24	70.96±0.92	91.95±1.58
**III** **e**	O	-CH(CH_3_)CH_2_N(CH_3_)_2_^ .^ HCl	86.92±1.41	85.28±0.67	73.06±2.04	73.97±1.31	129.12±1.72	92.62±0.97	88.13±0.77
**III** **f**	O	-CH(CH_3_)CH_2_N(C_2_H_5_)_2_^.^ HCl	94.43±0.75	76.83±1.19	78.84±0.35	80.64±0.94	87.47±1.90	84.55±1.68	93.70±1.54
**III** **g**	O	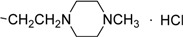	76.95±0.67	68.62±1.20	53.67±1.69	58.17±0.98	73.98±0.54	75.19±0.74	81.96±1.10
**III** **h**	O	-CH(CH_3_)CH_2_N(CH_3_)_2 _^.^ HCl	78.07±1.71	75.03±1.28	53.50±1.22	68.57±0.84	54.61±1.28	66.48±0.91	81.99±0.94
**IVa**	NH	-CH_2_CH_2_N(CH_3_)_2_^.^ HCl	< 10	< 10	< 10	< 10	< 10	< 10	< 10
**IV** **b**	NH	-CH_2_CH_2_N(C_2_H_5_)_2_^.^ HCl	63.80±1.87	91.73±1.01	71.70±1.35	58.78±0.86	87.11±0.87	82.59±1.23	126.86±2.90
**IV** **c**	NH	-(CH_2_)_3_N(CH_3_)_2_^.^ HCl	74.82±1.79	86.17±0.86	78.90±1.05	66.13±1.39	181.76±1.60	84.26±1.20	91.70±1.10
**IV** **d**	NH	-(CH_2_)_3_N(C_2_H_5_)_2_^.^ HCl	80.20±1.71	78.66±0.60	50.94±0.46	53.14±2.42	175.59±1.54	61.00±3.91	101.83±1.20
**IV** **e**	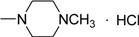		88.19±1.21	96.05±0.70	96.01±0.76	71.27±1.18	162.14±1.48	81.99±1.34	94.81±0.78
**IV** **f**	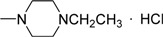		79.63±0.42	115.79±4.25	67.08±0.75	67.09±1.30	99.92±1.37	76.28±1.15	90.64±0.83
**IV** **g**	NH	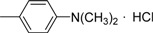	64.24±1.22	73.56±0.87	48.29±1.27	62.58±1.43	54.12±1.70	87.42±0.54	91.90±3.14
**IV** **h**	NH	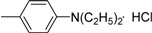	64.16±0.76	55.78±0.60	32.65±0.63	54.94±1.12	32.77±2.35	83.06±1.76	88.47±1.40
**IV** **i**	NH	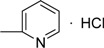	62.89±2.46	59.61±0.87	34.97±0.68	59.39±1.14	52.12±0.68	93.25±0.82	73.44±0.99
**I**	-OH		70.94±2.75	62.84±5.80	59.22±1.71	70.53±0.45	71.94±1.44	77.08±1.90	97.57±1.04
Tem	-NH_2_		85.13±2.97	68.51±1.25	62.35±0.54	86.85±1.67	70.67±1.02	85.20±0.93	76.03±1.38

^a.^ Survival percent (%) at 40 µg /mL of tested compounds on seven human tumor cell lines. Data shown are means ± SD of three independent experiments. Tem = temozolomide

In addition, the activity of partial synthetic compounds against leukemia cell line HL-60 was monitored by surveying the IC_50_ values compared with control compound temozolomide. The IC_50_ values of all tested compounds are listed in Table 2. The growth inhibition activity against HL-60 cell of compound **IIIa, IIIb, IIIc, IIId, IIIe, IIIf, IVf, IVg**, was more powerful than that of temozolomide. The IC_50_ values were 12.11 μmol/mL, 3.24 μmol/mL, 3.32 μmol/mL, 2.80 μmol/mL, 2.63 μmol/mL, 3.18 μmol/mL, 44.84 μmol/mL, 24.95 μmol/mL, and >80 μmol/mL, respectively, making them potentially promising candidates for the treatment of leukemia, Further antileukemia studies will be conducted.

**Table 2:** Growth inhibition activity of some temozolomide analogues on HL-60 cell.

Compd.	IC_50_(μmol/mL)^a)^	Compd.	IC_50_(μmol/mL)^a)^	Compd.	IC_50_(μmol/mL)^a)^
**IIIa**	12.11±1.21	**IVa**	>80	**IVg**	24.95±0.17
**IIIb**	3.24±0.09	**IVb**	>80	**IVh**	70.74±2.57
**IIIc**	3.32±0.16	**IVc**	>80	**IVi**	79.50±1.22
**IIId**	2.80±0.26	**IVd**	>80	**I**	45.47±0.35
**IIIe**	2.63±0.24	**IVe**	>80	**Tem**	>80
**IIIf**	3.18±0.07	**IVf**	44.84±0.30		

^a.^ Cells were treated with various concentrations of the tested compounds for 3 days and the cell growth inhibition was determined using the trypan blue assay. Data shown are means ± SD of three independent experiments.

As ring-opening of temozolomide and its derivatives can occur under basic or neutral conditions [8], the stability under different acidic conditions (pH 6.5, pH 6.0, pH 4.5, pH 3.6) was determined by high performance liquid chromatography (HPLC). After 20 min, more than 97% of all tested compounds remained intact at pH 3.6, whereas more than 20% of the compounds were decomposed under other pH conditions. The improvement of water-solubility of the synthesized compounds compared with temozolomide was estimated by calculating the HPLC peak area ratios in CH_3_COOH-CH_3_COONa buffer with pH 3.6. As shown in Table 3, the solubility of all compounds had increased from 10-fold to 100-fold. The solubility of compound **IIIa** – **IIIh**, **IVa** – **IVf** was increased at least 60-fold compared to that of temozolomide.

**Table 3:** The relative solubility of target compounds **IIIa** – **IVi** compared with temozolomide.

Compd.	A_1_/A_0_^ a)^	Compd.	A_1_/A_0_^ a)^	Compd.	A_1_/A_0_^ a)^
**IIIa**	88±2	**III** **g**	107±3	**IVe**	85±3
**IIIb**	97±4	**III** **h**	98±2	**IVf**	83±2
**IIIc**	78±2	**IVa**	76±2	**IVg**	35±3
**IIId**	93±2	**IVb**	64±4	**IVh**	36±4
**IIIe**	103±3	**IVc**	77±3	**IVi**	13±3
**IIIf**	91±3	**IVd**	62±3		

^a.^ Peak area ratio of synthesized compounds to temozolomide at pH 3.6. A_1_: the peak area of synthesized compound. A_0_: the peak area of temozolomide. Data shown are means ± SD of three independent experiments.

## 3. Experimental

### 3.1. General

^1^H-NMR spectra were recorded on a BRUKER ARX-300 instrument in (CD_3_)_2_SO solution with Me_4_Si as internal standard. MS were determined on Shimadzu GCMS QP-1000 mass spectrometer. HR-MS were obtained on Finnigan MAT-711 mass spectrometer in EI mode. Infrared spectra were recorded on a BRUKER IFS-55 FTIR spectrometer. The purity, stability and water-solubility were calculated in a N300 chromatographic workstation equipped with a Hitachi L-2400 UV detector and Hitachi pump-L-2130. Optical rotation was recorded on Pekin-Elmer 241 instrument. Melting points were determined on a Yanaco melting point apparatus and are uncorrected. Unless specified otherwise, all reagents and solvents were used as supplied by the manufacturer. Temozolomide and compound **I** were synthesized according to the literature [8]. Disubstituted aminoalkyl alcohols and disubstituted aminoalkyl amines were synthesized according to the literature [9,10].

*3-Methyl-4-oxo-3,4-dihydroimidazo[5,1-d][1,2,3,5]tetrazine-8-acyl chloride* (**II**). A mixture of compound **I** (2.0 g, 0.01 mol), SOCl_2_ (20 mL) and DMF (2 drops) was refluxed 2.5 h, then evaporated under reduced pressure. Toluene (10 mL) was added, and the solution was again evaporated to dryness to give **II** as a light yellow powder (1.9 g, 97.4%); m.p. 142-143 ºC.

*General procedure for the synthesis of target compounds* **IIIa-IIIh, IVa-Ivi**. The appropriate disubstituted aminoalkyl alcohol or disubstituted aminoalkyl amine (0.00256 mol) was added dropwise at room temperature to a solution of **II** (0.5 g, 0.00256 mol) in anhydrous THF (20 mL). The mixture was reacted 3 h, and the precipitate that appeared was filtered and washed with anhydrous THF, ether, and recrystalled from acetone-methanol to give the title compounds.

*[2-(N,N-Dimethylamino)]ethyl 3-methyl-4-oxo-3,4-dihydroimidazo[5,1-d][1,2,3,5]tetrazine-8 carb- oxylate hydrochloride* (**IIIa**). Yield 54.5%; m.p.: 208-209 ºC; purity: 98.1%; IR (KBr): 3445, 2974, 2723, 1774, 1741, 1619, 1465, 1230 cm^-1^; MS [M^+^] (*m/z*): 266; ^1^H-NMR (DMSO-d_6_) δ: 2.89 (s, 6H, N(CH_3_)_2_), 3.54 (t, 2H, CH_2_CH_2_N), 3.90 (s, 3H, 3-CH_3_), 4.73 (t, 2H, OCH_2_- CH_2_), 8.93 (s, 1H, 6-CH), 10.74 (s, 1H, HCl); HR-MS: *m/z* calcd. for C_10_H_14_N_6_O_3_, (M^+^) 266.1127. Found: 266.1130. 

*[2-(N,N-Diethylamino)]ethyl 3-methyl-4-oxo-3,4-dihydroimidazo[5,1-d][1,2,3,5]tetrazine-8-carb-oxylate hydrochloride* (**IIIb**). Yield 53.2%; m.p.: 208-209 ºC; purity: 97.9%; IR (KBr): 3432, 3038, 2951, 2593, 1758, 1728, 1247 cm^-1^; MS [M^+^] (*m/z*): 294; ^1^H-NMR (DMSO-d_6_) δ: 1.25 (t, *J* = 7.2 Hz, 6H, N(CH_2_CH_3_)_2_), 3.29 (q, *J* = 7.2 Hz, 4H, N(CH_2_CH_3_)_2_), 3.54 (t, 2H, OCH_2_CH_2_N), 3.90 (s, 3H, CH_3_), 4.73 (t, 2H, OCH_2_CH_2_), 8.93 (s, 1H, 6-CH), 10.57 (brs, 1H, HCl); HR-MS: *m/z* calcd. for C_12_H_18_N_6_O_3_, (M^+^) 294.1440. Found: 294.1428.

*[3-(N,N-Dimethylamino)]propyl 3-methyl-4-oxo-3,4-dihydroimidazo[5,1-d][1,2,3,5]tetrazine-8-carb-oxylate hydrochloride* (**IIIc**). Yield 54.3%; m.p.: 224-225 ºC; purity: 98.5%; IR (KBr): 3461, 3074, 2960, 2599, 1750, 1723, 1462, 1248 cm^-1^; MS [M^+^] (*m/z*): 280; ^1^H-NMR (DMSO-d_6_) δ: 2.16 (m, 2H, CH_2_CH_2_CH_2_), 2.79 (s, 6H, N(CH_3_)_2_), 3.23 (t, *J* = 7.4 Hz, 2H, CH_2_CH_2_N), 3.89 (s, 3H, 3-CH_3_), 4.42 (t, *J* = 6.1 Hz, 2H, OCH_2_CH_2_), 8.89 (s, 1H, 6-CH); HR-MS: *m/z* calcd. for C_11_H_16_N_6_O_3_, (M^+^) 280.1284. Found: 280.1281.

*[3-(N,N-Diethylamino)]propyl 3-methyl-4-oxo-3,4-dihydroimidazo[5,1-d][1,2,3,5]tetrazine-8-carb-oxylate hydrochloride* (**IIId**). Yield 56.8%, m.p.: 204-205 ºC, purity: 98.6%, IR (KBr): 3426, 3038, 2954, 2596, 1749, 1728, 1458, 1248 cm^-1^; MS [M^+^] (*m/z*): 308. ^1^H-NMR (DMSO-d_6_) δ: 1.22 (t, *J* = 7.3 Hz, 6H, N(CH_2_CH_3_)_2_), 2.13 (m, 2H, CH_2_CH_2_CH_2_), 3.11 (q, *J* = 7.3 Hz, 4H, N(CH_2_CH_3_)_2_), 3.19 (t, 2H, CH_2_CH_2_N), 3.87 (s, 3H, 3-CH_3_), 4.42 (t, *J* = 5.9 Hz, 2H, OCH_2_CH_2_), 8.88 (s, 1H, 6-CH), 10.45 (s, 1H, HCl). HR-MS: *m/z* calcd. for C_13_H_20_N_6_O_3_, (M^+^) 308.1597. Found: 308.1608.

*[1-Methyl-2-(N,N-dimethylamino)]ethyl 3-methyl-4-oxo-3,4-dihydroimidazo[5,1-d][1,2,3,5]tetrazine-8-carboxylate hydrochloride* (**IIIe**). Yield 68.5%; m.p.: 202-203 ºC; purity: 99.0%; IR (KBr): 3504, 3127, 2956, 2632, 1749, 1733, 1459, 1243 cm^-1^; MS [M^+^] (*m/z*): 280; ^1^H-NMR (DMSO-d_6_) δ: 1.39 (d, *J* = 6.3 Hz, 3H, OCHCH_3_), 2.86 (s, 6H, N(CH_3_)_2_), 3.47 (d, J = 8.5 Hz, 2H, CHCH_2_N), 3.89 (s, 3H, 3-CH_3_), 5.54 (m, 1H, OCHCH_3_), 8.90 (s, 1H, 6-CH); HR-MS: *m/z* calcd. for C_11_H_16_N_6_O_3_, (M^+^) 280.1284. Found: 280.1281.

*[1-Methyl-2-(N,N-diethylamino)]ethyl 3-methyl-4-oxo-3,4-dihydroimidazo[5,1-d][1,2,3,5]tetrazine-8-carboxylate hydrochloride* (**IIIf**). Yield 68.3%; m.p.: 194-195 ºC; purity: 98.5%; IR (KBr): 3443, 3130, 2975, 2471, 1750, 1728, 1458, 1243 cm^-1^; MS [M^+^] (*m/z*): 308; ^1^H-NMR (DMSO-d_6_) δ: 1.26 (q, *J* = 7.3 Hz, 6H, CH_2_CH_3_), 1.41 (d, *J* = 6.2 Hz, 3H, OCHCH_3_), 3.21 (q, *J* = 7.3 Hz, 4H, CH_2_CH_3_), 3.50 (d, *J* = 4.6 Hz, 2H, CHCH_2_N), 3.89 (s, 3H, 3-CH_3_), 5.50 (m, 1H, OCHCH_3_), 8.89 (s, 1H, 6-CH), 10.08 (s, 1H, HCl); HR-MS: *m/z* calcd. for C_13_H_20_N_6_O_3_, (M^+^) 308.1597. Found: 308.1591.

*[2-(4-Methylpiperazine)-1-]ethyl 3-methyl-4-oxo-3,4-dihydroimidazo[5,1-d][1,2,3,5]tetrazine-8-carb-oxylate hydrochloride* (**IIIg**). Yield 68.5%; m.p.: 183-185 ºC; purity: 97.8%; IR (KBr): 3426, 3121, 2957, 2585, 1743, 1720, 1463, 1249 cm^-1^; MS [M^+^] (*m/z*): 321; ^1^H-NMR (DMSO-d_6_) δ: 3.89 (s, 3H, 3-CH_3_), 4.46 (t, 2H, OCH_2_CH_2_), 8.86 (s, 1H, 6-CH), 10.21 (brs, 1H, HCl); HR-MS: *m/z* calcd. for C_13_H_19_N_7_O_3_, (M^+^) 321.1549. Found: 321.1544.

*(-)-[1-Methyl-2-(N,N-dimethylamino)]ethyl 3-methyl-4-oxo-3,4-dihydroimidazo[5,1-d][1,2,3,5] tet-razine-8-carboxylate hydrochloride* (**IIIh**). Yield 75.1%; m.p.: 208-209 ºC; [α]_D_^25^ = -14.7º; purity: 99.1%; IR (KBr): 3430, 3127, 2958, 2629, 1749, 1720, 1461, 1245 cm^-1^; MS [M^+^] (*m/z*): 280; ^1^H-NMR (DMSO-d_6_) δ: 1.38 (d, *J* = 6.3 Hz, 3H, OCHCH_3_), 2.86 (s, 6H, N(CH_3_)_2_), 3.49 (d, *J* = 8.6 Hz, 2H, CHCH_2_N), 3.89 (s, 3H, 3-CH_3_), 5.54 (m, 1H, OCHCH_3_), 8.89 (s, 1H, 6-CH), 10.36 (brs, 1H, HCl); HR-MS: *m/z* calcd. for C_11_H_16_N_6_O_3_, (M^+^) 280.1284. Found: 280.1297.

*3-Methyl-4-oxo-3,4-dihydroimidazo[5,1-d][1,2,3,5]tetrazine-8-[N-(2-dimethylamino-)ethyl] carbox-amide hydrochloride* (**IVa**). Yield 75.3%; m.p.: 180-181 ºC; purity: 97.6%; IR (KBr): 3387, 3113, 2678, 1747, 1659, 1575, 1460, 1205 cm^-1^; MS [M^+^] (*m/z*): 265; ^1^H-NMR (DMSO-d_6_) δ: 2.82 (s, 6H, N(CH_3_)_2_), 3.39 (t, *J* = 6.0 Hz, 2H, CH_2_CH_2_N), 3.68 (t, *J* = 6.0 Hz, 2H, CNHCH_2_CH_2_), 3.88 (s, 3H, 3-CH_3_), 8.81 (t, *J* = 5.6 Hz, 1H, CONH), 8.89 (s, 1H, 6-CH), 10.09 (brs, 1H, HCl); HR-MS: *m/z* calcd. for C_10_H_15_N_7_O_2_, (M^+^) 265.1287. Found: 265.1303.

*3-Methyl-4-oxo-3,4-dihydroimidazo[5,1-d][1,2,3,5]tetrazine-8-[N-(2-diethylamino)ethyl]carboxamide hydrochloride* (**IVb**). Yield 63.2%; m.p.: 118-119 ºC; purity: 97.7%; IR (KBr): 3460, 3234, 3102, 2940, 2643, 1739, 1656, 1255 cm^-1^; MS [M^+^] (*m/z*): 293; ^1^H-NMR (DMSO-d_6_) δ: 1.22 (t, *J* = 7.2 Hz, 6H, CH_2_CH_3_), 3.66 (m, 2H, CONHCH_2_CH_2_), 3.87 (s, 3H, 3-CH_3_), 8.82 (t, *J* = 5.7 Hz, 1H, CONH), 8.90 (s, 1H, 6-CH); HR-MS: *m/z* calcd. for C_12_H_19_N_7_O_2_, (M^+^) 293.1600. Found: 293.1633.

*3-Methyl-4-oxo-3,4-dihydroimidazo[5,1-d][1,2,3,5]tetrazine-8-[N-(3-dimethylamino)propyl] carbox-amide hydrochloride* (**IVc**). Yield 67.4%; m.p.: 117-119 ºC; purity: 97.5%; IR (KBr): 3409, 3266, 2959, 2644, 1756, 1647, 1576, 1258 cm^-1^; MS [M^+^] (*m/z*): 279; ^1^H-NMR (DMSO-d_6_) δ: 1.92 (m, 2H, CH_2_CH_2_CH_2_), 2.75 (s, 6H, N(CH_3_)_2_), 3.07 (t, *J* = 7.7 Hz, 2H, CH_2_CH_2_N), 3.38 (t, *J* = 6.4 Hz, 2H, CONHCH_2_), 3.87 (s, 3H, 3-CH_3_), 8.72 (t, 1H, CONH), 8.87 (s, 1H, 6-CH), 10.06 (brs, 1H, HCl); HR-MS: *m/z* calcd. for C_11_H_17_N_7_O_2_, (M^+^) 279.1444. Found: 279.1430.

*3-Methyl-4-oxo-3,4-dihydroimidazo[5,1-d][1,2,3,5]tetrazine-8-[N-(3-diethylamino)propyl] carbox-amide hydrochloride* (**IVd**). Yield 65.1%; m.p.: 182-183 ºC; purity: 98.0%; IR (KBr): 3442, 3252, 2949, 2648, 1733, 1652, 1246 cm^-1^; MS [M^+^] (*m/z*): 307; ^1^H-NMR (DMSO-d_6_) δ: 1.19 (t, *J* = 7.2 Hz, 6H, CH_2_CH_3_), 1.92 (m, 2H, CH_2_CH_2_CH_2_), 3.39 (t, *J* = 5.8 Hz, 2H, CONHCH_2_), 3.87 (s, 3H, 3-CH_3_), 8.73 (t, 1H, CONH), 8.87 (s, 1H, 6-CH), 10.06 (brs, 1H, HCl); HR-MS: *m/z* calcd. for C_13_H_21_N_7_O_2_, (M^+^) 307.1757. Found: 307.1758.

*1-(3-Methyl-4-oxo-3,4-dihydroimidazo[5,1-d][1,2,3,5]tetrazine-8-oyl)-4-methyl piperazine hydro-chloride* (**IVe**). Yield 71.8%; m.p.: 189-190 ºC; purity: 97.9%; IR (KBr): 3434, 3059, 2943, 2453, 1758, 1646 cm^-1^; MS [M^+^] (*m/z*): 277; ^1^H-NMR (DMSO-d_6_) δ: 2.75 (s, 3H, NCH_3_), 3.21 (m, 8H, N(CH2CH_2_)_2_N), 3.85 (s, 3H, 3-CH_3_), 8.88 (s, 1H, 6-CH); HR-MS: *m/z* calcd. for C_11_H_15_N_7_O_2_, (M^+^) 277.1287. Found: 277.1287.

*1-(3-Methyl-4-oxo-3,4-dihydroimidazo[5,1-d][1,2,3,5]tetrazine-8-oyl)-4-ethyl piperazine hydro-chloride* (**IVf**). Yield 70.8%; m.p.: 200-201 ºC; purity: 98.3%; IR (KBr): 3435, 3119, 2361, 1746, 1656, 1558, 1460, 1242 cm^-1^; MS [M^+^] (*m/z*): 291; ^1^H-NMR (DMSO-d_6_) δ: 1.26 (t, *J* = 7.1 Hz, 3H, CH_2_CH_3_), 3.87 (s, 3H, 3-CH_3_), 8.89 (s, 1H, 6-CH); HR-MS: *m/z* calcd. for C_12_H_17_N_7_O_2_, (M^+^) 291.1444. Found: 291.1453.

*3-Methyl-4-oxo-3,4-dihydroimidazo[5,1-d][1,2,3,5]tetrazine-8-[N-(p-dimethylamino)phenyl] carbox-amide hydrochloride* (**IVg**). Yield 70.7%; m.p.: 209-210 ºC; purity: 98.1%; IR (KBr): 3347, 3054, 2208, 1758, 1679, 1608 cm^-1^; MS [M^+^] (*m/z*): 313; ^1^H-NMR (DMSO-d_6_) δ: 3.04 (s, 6H, N(CH_3_)_2_ ), 3.89 (s, 3H, 3-CH_3_ ), 7.33 (brs, 2H, 3’-, 5’-CH (Ph) ), 7.89 (brs, 2H, 2’-, 6’-CH(Ph) ), 8.96 ( s, 1H, 6-CH ), 10.45 ( brs, 1H, HCl ); HR-MS: *m/z* calcd. for C_14_H_15_N_7_O_2_, (M^+^) 313.1287. Found: 313.1268.

*3-Methyl-4-oxo-3,4-dihydroimidazo[5,1-d][1,2,3,5]tetrazine-8-[N-(p-diethylamino)phenyl] carbox-amide hydrochloride* (**IVh**). Yield 67.8%; m.p.: 215-216 ºC; purity: 97.7%; IR (KBr): 3362, 3119, 2309, 1748, 1690, 1606, 1573, 1251 cm^-1^; MS [M^+^] (*m/z*): 341; ^1^H-NMR (DMSO-d_6_) δ: 1.04 (t, *J* = 6.9 Hz, 6H, (CH_2_CH_3_)_2_), 3.42 (q, *J* = 6.9 Hz, 4H, (CH_2_CH_3_)_2_), 3.90 (s, 3H, 3-CH_3_), 7.72 (brs, 2H, 3’-, 5’-CH Ph), 8.10 (brs, 2H, 2’-, 6’-CHPh), 8.98 (s, 1H, 6-CH), 10.74 (brs, 1H, HCl), 11.91 (brs, 1H, CONH); HR-MS: *m/z* calcd. for C_16_H_19_N_7_O_2_, (M^+^) 341.1600. Found: 341.1608.

*3-Methyl-4-oxo-3,4-dihydroimidazo[5,1-d][1,2,3,5]tetrazine-8-(N-pyridine-2-)carboxamide hydro- chloride* (**IVi**). Yield 65.4%; m.p.: 217-218 ºC; purity: 98.5%; IR (KBr): 3358, 3096, 1737, 1642, 1619, 1569, 1239 cm^-1^; MS [M^+^] (*m/z*): 271; ^1^H-NMR (DMSO-d_6_) δ: 3.89 (s, 3H, 3-CH_3_), 7.25 (t, *J* = 7.0 Hz, 1H, Pyr-5-H), 7.95 (t, *J* = 7.0 Hz, 1H, Pyr-4-H), 8.24 (d, *J* = 8.3 Hz, 1H, Pyr-6-H), 8.41, (d, *J* = 5.0 Hz, 1H, Pyr-3-H), 8.79 (s, 1H, CONH), 8.97 (s, 1H, 6-CH), 10.14 (s, 1H, HCl); HR-MS: *m/z* calcd. for C_11_H_9_N_7_O_2_, (M^+^) 271.0818. Found: 271.0811.

### 3.2. Biological activity assays

*MTT assay*: 10^3^ cells were seeded in RPMI1640 in each well of a 96-well plate and were allowed to adhere and spread at 37 ºC, 5% CO_2_ for 24 h. The compounds with the concentration of 40 μg/mL were then added and incubated for 4 d. Fifty μL of 2 mg/mL MTT solution was added per well and the cultures were continued for an additional 4 h. The medium was removed by aspiration. The cells were dissolved in 200 μL DMSO and vibrated for 10 min. Absorbance at 540 nm was measured in the 96-well plate. Growth inhibition was determined as compared to untreated cells (%).

*Trypan Blue assay*: Cells were seeded at a density of 1 × 10^5^ cells/mL and incubated with various concentrations of the tested compounds for 3 days. Total cell number including trypan blue staining positive and negative cells in each group was counted. The cell growth inhibition ability was calculated and expressed as the ratio of the cell number in treated group to that of untreated group. The concentration (IC_50_) which inhibited half of the cell growth was calculated.

### 3.3. Solubility detection

Compound and temozolomide were dissolved in sodium acetate - acetic acid buffer solution (pH 3.6) to form a supersaturated solution. The suspension was shaken by ultrasonic irradiation, and filtered. The peak area ratio was calculated by HPLC with UV detection at 254 nm.

## 4. Conclusions

In summary, the results presented above indicate that 1) temozolomide esters are more effective than temozolomide amides in inhibiting HL-60 cell growth, 2) amide **IVa** with 2-dimethylamino-ethyl chain has the most powerful potential in all tested solid tumor cell lines; 3) the water-solubility of all synthesized compounds has been improved in various degrees.
